# Risk of myocardial infarction (MI) and death following MI in people with chronic obstructive pulmonary disease (COPD): a systematic review and meta-analysis

**DOI:** 10.1136/bmjopen-2015-007824

**Published:** 2015-09-11

**Authors:** Kieran J Rothnie, Ruoling Yan, Liam Smeeth, Jennifer K Quint

**Affiliations:** 1 Respiratory Epidemiology, Occupational Medicine and Public Health, National Heart and Lung Institute, Imperial College London, London, UK; 2 Faculty of Epidemiology and Population Health, London School of Hygiene & Tropical Medicine, London, UK; 3 Medical School, Faculty of Medical Sciences, University College London, London, UK

## Abstract

**Objectives:**

Cardiovascular disease is an important comorbidity in patients with chronic obstructive pulmonary disease (COPD). We aimed to systematically review the evidence for: (1) risk of myocardial infarction (MI) in people with COPD; (2) risk of MI associated with acute exacerbation of COPD (AECOPD); (3) risk of death after MI in people with COPD.

**Design:**

Systematic review and meta-analysis.

**Methods:**

MEDLINE, EMBASE and SCI were searched up to January 2015. Two reviewers screened abstracts and full text records, extracted data and assessed studies for risk of bias. We used the generic inverse variance method to pool effect estimates, where possible. Evidence was synthesised in a narrative review where meta-analysis was not possible.

**Results:**

Searches yielded 8362 records, and 24 observational studies were included. Meta-analysis showed increased risk of MI associated with COPD (HR 1.72, 95% CI 1.22 to 2.42) for cohort analyses, but not in case–control studies: OR 1.18 (0.80 to 1.76). Both included studies that investigated the risk of MI associated with AECOPD found an increased risk of MI after AECOPD (incidence rate ratios, IRR 2.27, 1.10 to 4.70, and IRR 13.04, 1.71 to 99.7). Meta-analysis showed weak evidence for increased risk of death for patients with COPD in hospital after MI (OR 1.13, 0.97 to 1.31). However, meta-analysis showed an increased risk of death after MI for patients with COPD during follow-up (HR 1.26, 1.13 to 1.40).

**Conclusions:**

There is good evidence that COPD is associated with increased risk of MI; however, it is unclear to what extent this association is due to smoking status. There is some evidence that the risk of MI is higher during AECOPD than stable periods. There is poor evidence that COPD is associated with increased in hospital mortality after an MI, and good evidence that longer term mortality is higher for patients with COPD after an MI.

**Strengths and limitations of this study**

- This systematic review investigated three important areas relating to the relationship between chronic obstructive pulmonary disease (COPD) and cardiovascular disease: (1) the risk of myocardial infarction (MI) associated with COPD; (2) the risk of MI associated with acute exacerbations of COPD; and (3) the risk of death following MI in patients with COPD compared to patient without COPD.
- Strengths of this review were the wide search strategy, broad inclusion criteria and rigorous risk of bias assessment of included studies.
- We found strong evidence for an increased risk of MI in people with COPD and an increased risk of longer term death after MI for patients with COPD; however, it is unclear how much of this increased risk may be due to smoking status.
- We found poorer evidence for an increased risk of MI during periods of acute exacerbation of COPD compared to stable periods, and for an increased risk of death in hospital after MI for patients with COPD. We make recommendations on how future studies can improve our understanding of these relationships.
- Due to statistical and clinical heterogeneity, meta-analysis could only be conducted for some of the research questions.

## Introduction

Cardiovascular disease is a common comorbidity and cause of death in people with chronic obstructive pulmonary disease (COPD), with up to one-third dying of cardiovascular disease.^1^ Reducing the cardiovascular disease in this population is an important strategy for reducing the burden of COPD.

Several studies have shown that people with COPD have a higher risk of myocardial infarction (MI) than people without COPD.^2–4^ One of the reasons for the increased risk of MI in patients with COPD is the shared major risk factor of smoking. In addition, several other cardiovascular risk factors, including hypertension, diabetes, inactivity, poor diet, and older age, are also prevalent in patients with COPD.^5–7^ In addition, several studies have found an association between reduced FEV_1_ (forced expiratory volume1 s) and cardiovascular mortality in the general population.^8^ However, COPD itself is also thought to be an independent risk factor for MI with increased risk of MI possibly being mediated through increased systemic inflammation or reduced FEV_1_ in people with COPD.

Acute exacerbations of COPD are events in the natural history of COPD which are characterised by an increase in COPD symptoms such as breathlessness, cough, sputum volume, and sputum purulence. It has recently been suggested that acute exacerbations of COPD (AECOPD) represent a period of increased risk of MI for people with COPD.^9^ A subtype of patients with COPD appears to have more frequent exacerbations than others. Frequent exacerbators have been defined as individuals who have two or more treated exacerbations per year. Frequent exacerbators may be at higher risk of MI compared to infrequent exacerbators, even during stable periods.

Several investigators have found that patients with COPD have worse mortality in hospital and following discharge after an MI compared to patient without COPD.^10–12^ However, the finding that patients with COPD have greater in hospital and short-term mortality has not been found by all investigators.^13–15^

We aimed to systematically review the literature reporting on: (1) the risk of MI in people with COPD; (2) the risk of MI associated AECOPD, either during AECOPD or that associated with the frequent exacerbator phenotype; and (3) the risk of death after MI in people with COPD. These questions represent the most salient aspects of current research into the relationship between COPD and cardiovascular disease, and no systematic reviews have been published on these topics to date.

## Methods

### Literature search

MEDLINE, MEDLINE In-Process & Other Non-Indexed Citations, EMBASE, BIOSIS & Science Citation Index were searched up to January 2015. A search strategy was devised which would pick up articles relevant to all three research questions. All strategies were based on the MEDLINE search strategy, which is presented in the online supplementary material. In brief, the literature was searched for terms which relate to COPD and terms with relate to MI, and these searches were combined using the AND Boolean logic operator. MeSH terms were combined with natural language searching using truncation where appropriate.

### Inclusion and exclusion criteria

Inclusion and exclusion criteria were applied for each of the three research questions. Studies were included if they met the population, exposure, comparator and outcome criteria. These are presented below for each research question. Studies were included from database start date and were not restricted by language.

#### Risk of MI in people with COPD

The population of interest was the general population. The exposure of interest was diagnosis of COPD. The un-exposed group was people without a diagnosis of COPD. The outcome of interest was acute MI.

#### Risk of MI associated with AECOPD

The population of interest was people with a diagnosis of COPD. The exposures of interest were either: (1) discrete episodes of AECOPD or periods within 8 weeks of an AECOPD; or (2) frequent exacerbator phenotype. The comparators of interest were either: (1) periods of stable COPD; or (2) infrequent exacerbator phenotype. Studies were included if these reported a relative risk of MI, or if this could be calculated.

#### Risk of death after MI in people with COPD

The population of interest was those presenting to a hospital with an MI. Studies were included if these compared patients with a diagnosis of COPD to those without a diagnosis of COPD. Outcomes of interest were death in hospital and at any reported time points post discharge. Studies investigating risk of death for patients with COPD after an interventional procedure following an MI (such as percutaneous coronary intervention or coronary artery bypass graft) were specifically excluded under the population criterion.

### Selection of included studies

Titles and abstracts, where available, were initially screened for potential inclusion by one reviewer. Full text versions of potentially included studies were then obtained and were screened by two reviewers. Authors were contacted if the information provided in articles was not sufficient to assess whether inclusion criteria were satisfied.

### Risk of bias assessment

All included studies, except for those only reported as conference abstracts, were assessed for risk of bias. The risk of bias tool was informed by the Newcastle-Ottawa scale;^16^ however, we did not make use of a summary score as this is not advisable.^17^ ^18^ Risk of bias was assessed across the key domains related to selection of participants, comparability of groups, and measurement of outcomes. Several items were included under each domain, and were adapted for different study types. Where reports of studies included more than one analysis (eg, a case control as well as a cohort analysis) the risk of bias for these analyses were conducted separately. Risk of bias assessment was completed by one reviewer and checked by another.

### Evidence synthesis

Characteristics and findings of studies were tabulated and compared. Data on severity of COPD was extracted as GOLD stage or FEV_1_% predicted, where available. Information was also extracted on smoking status and previous cardiovascular disease. Estimates of effect were extracted or calculated, and are presented as ORs, risk ratios (RR), incidence rate ratios (IRR) or HRs.

Where included studies were reasonably statistically and clinically similar, we pooled results using random effects meta-analysis. We used the generic inverse variance method to pool maximally adjusted effect estimates. Analysis was conducted in Review Manager 5.3. Where studies were too statistically (I^2^ over 75%) or clinically heterogeneous, meta-analysis was not conducted, but study summary results were graphed on forest plots without pooling the results. Studies which were not adjusted at all were not included in forest plots. For the question on risk of MI associated with COPD, studies were stratified by adjustment for smoking status (yes or no) and study design (cohort or case–control). For the question on risk of death following MI in COPD compared to patient without COPD, studies were stratified by outcome time point (in-hospital mortality or follow-up mortality). For follow-up mortality, studies were further stratified by analysis.

## Results

### Identified studies

**Figure 1:**
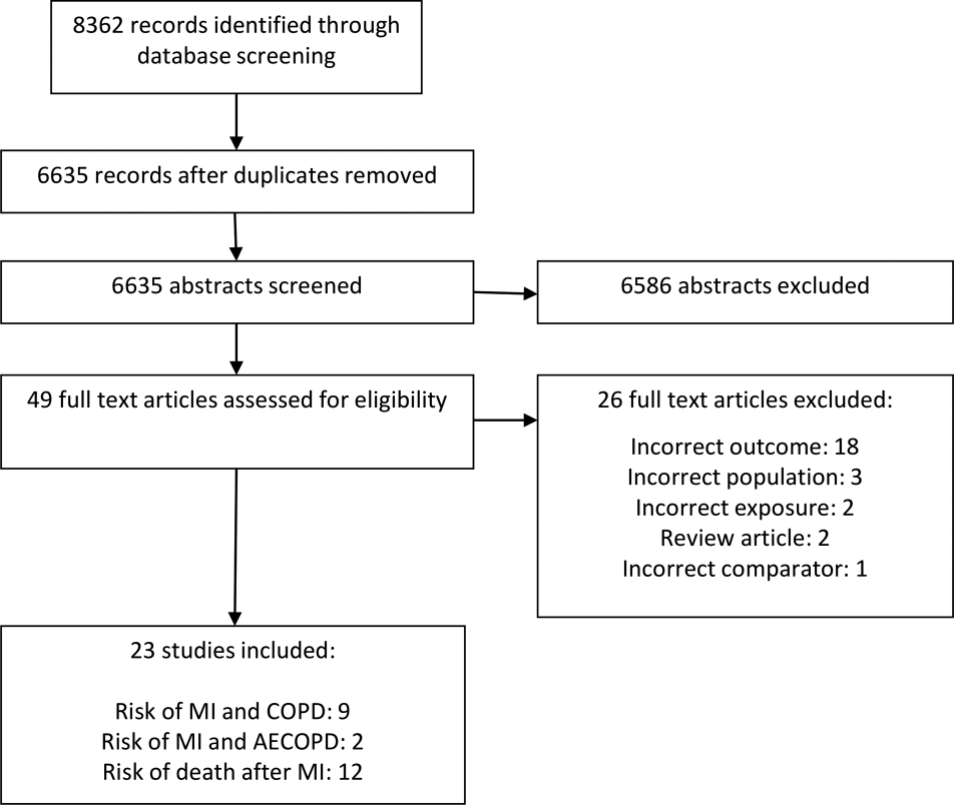
Study selection. AECOPD, acute exacerbation of COPD; COPD, chronic obstructive pulmonary disease; MI, myocardial infarction.

Literature searches yielded 8362 records. After title and abstract screening, 49 records were selected for full-text assessment, which resulted in the inclusion of 24 studies. The inclusion and exclusion process is summarised in figure 1. Of the 24 included studies, 9 investigated the risk of MI in patients with COPD compared to patient without COPD; 2 investigated the risk of MI associated with AECOPD; no studies were found which investigated the risk of MI associated with the frequent exacerbator phenotype; and 12 investigated outcomes after MI for patients with COPD compared to patient without COPD. Summary characteristics of included studies are presented in tables 1–3.

**Table 1.** Characteristics of included studies – risk of MI associated with COPD

Study	Design and setting	Population	Characteristics of COPD patients	MI definition	Maximally adjusted estimate (95% CI)	Factors adjusted for
Curkendall *et al*^ 2^ ^30^	Cohort in the Saskatchewan Health databases 1998–2001.	11 493 COPD patients ≥40 years, identified by physician claim or hospital discharge COPD code and at least two prescriptions for COPD medicines within 6 months of the index COPD code. 22 986 age and sex matched non-COPD patients	*Age* NR *Sex* NR *COPD severity* NR *Current smokers* 16% *History of CVD* Previous MI—2.3% Previous angina—6.6%	*Any MI during follow up:* any inpatient or outpatient diagnosis of MI *Hospitalisation due to MI:* primary hospital discharge diagnosis of MI *Fatal MI:* underlying cause of death which initiated the sequence of events that lead to death recorded as MI	*Any MI during follow up (period prevalence):* OR 1.61 (1.43–1.81) *Hospitalisation due to MI:* IRR 1.49 (0.71–3.13) *Fatal MI:* IRR 1.51 (1.14–2.01)	*Period prevalence of MI:* Age, sex, history of cardiovascular disease, diabetes, hypertension, hypercholesterolaemia *Hospitalisation for MI:* adjusted for history of cardiovascular events, diabetes, hypertension, and hypercholesterolemia using Poisson regression, age and sex by matching. *Fatal MI:* age and sex by matching only.
Feary *et al*^ 3^	Cohort in The Health Improvement Network, 2005–2007	29 870 COPD patients >35 years identified by COPD diagnostic code. 1 174 240 non-COPD patients	*Age* 35–44–1.8% 45–54–7.0% 55–64–20.5% 65–74–31.7% ≥75–39.0% *Sex* 48.1% male *COPD severity* FEV_1_ % predicted 50–80%–37.5% 30–49%–19.1% <30%–5.3% *Current smokers* 65.3% *History of CVD* Prior CVD–28.0%	Diagnostic code for MI in primary care record	*35–44 years:* HR 10.34 (3.28–32.6) *45–54 years:* HR 1.22 (0.55–2.74) *55–64 years:* HR 1.55 (1.07–2.26) *65–74 years:* HR 1.78 (1.37–2.31) ≥*75 years:* 1.34 (1.03–1.73)	Age, sex and smoking status
Huiart *et al*^ 28^	Cohort in the Saskatchewan Health databases 1990–1999	5 648 COPD patients ≥50 years, identified by prescription of three or more bronchodilators within the period of one year. Rates of MI compared to those of general Saskatchewan population	*Age* NR *Sex* NR *COPD severity* NR *Current smokers* NR *History of CVD* NR Characteristics were not split by COPD status.	Primary hospital discharge diagnosis of MI	*Standardised IRR:* 1.30 (1.15–1.44)	Age and sex by standardisation
Mapel *et al*^ 28^	Cohort in the Veterans Administration Medical System, 1991–1999	COPD patients identified by discharge codes (1991–1999) and/or outpatient codes (1997–1999) Age and sex matched controls without COPD	*Age* Median 60 (IQR, 49–62) *Sex* 95.7% male *COPD severity* NR *Current smokers* NR *History of CVD* Cardiovascular disease—71.2%	Specific ICD-9-CM code for MI during 1999 that was not present in 1998	*COPD patients identified using discharge codes* *IRR:* 1.28 (1.18–1.38) *COPD patients identified using outpatient codes* *IRR:* 5.31 (4.54–6.21)	Age and sex by matching
Rodriguez *et al*^ 19^	Cohort and case-control study in the General Practice Research Database, 1996–2001	1532 patients with a first COPD diagnosis in 1996, and no history of cardiovascular disease 13 500 age and sex matched non-COPD patients, with no history of cardiovascular disease	*Age* NR *Sex* NR *COPD severity* NR *Current smokers* NR *History of CVD* NR	Diagnostic code for MI in primary care record	*Cohort analysis:* IRR 1.18 (0.81–1.71) *Case-control analysis:* OR 0.93 (0.62–1.39)	*Cohort analysis:* Age and sex *Case control analysis:* Age, sex, smoking and number of primary care physician visits
Schneider *et al*^ 4^	Cohort and nested case-control study in the General Practice Research Database, 1995–2005	35 772 patients with a first COPD diagnosis between 1995–2005 35 772 non-COPD patients matched on age, sex and calendar time and general practice	*Age* 40–49–6.8% 50–59–19.9% 60–69–33.8% >70–39.6% *Sex* 51.3% male *COPD severity* NR *Current smokers* 43.3% *History of CVD* Prior MI/CHD—18.3% Prior CHF—8.4%	Diagnostic code for MI along with death or hospitalisation within 30 days of the diagnosis; and/or start of new treatment with ACE antagonist, β-blocker, statin, vitamin K antagonist, platelet aggregation inhibitor or aspirin within 90 days of the diagnosis in primary care record	*Cohort analysis:* IRR 1.56 (1.43–1.75) *Case control analysis:* Any COPD : OR 1.40 (1.13–1.73) Mild COPD: OR 1.79 (1.12–2.86) Moderate COPD: OR 1.30 (1.04–1.62) Severe COPD: OR 3.00 (1.53–5.86)	Cohort analysis: matched on age, sex, calendar time and general practice Case-control analysis: Smoking status, BMI, hypertension, hyperlipidaemia, diabetes and NSAID use
Sidney *et al*^ 29^	Cohort in health insurance database. North Carolina, 1996–1999	COPD defined as: hospitalisation or outpatient diagnosis of COPD, two or more prescriptions for COPD medicines, aged over 40 years. Non-COPD patients matched on age, sex and length of care plan membership.	*Age* 40–59–35% 60–79–55% >80–10% *Sex* 55.4% male *COPD severity* NR *Current smokers* NR *History of CVD* Prior MI—1.8% Prior angina—1.0% Prior CHF—7.2%	ICD code for acute MI	*Overall:* IRR 1.89 (1.71–2.09) *Men:* IRR 1.77 (1.56–2.01) *Women:* IRR 2.09 (1.78–2.46) *40–64 years:* IRR 2.43 (1.98–2.98) ≥*65 years:* IRR 1.73 (1.54–1.94)	Age, sex and baseline cardiovascular risk profile.
Sode *et al*^ 31^	Cohort study within the National Danish patient registry, 1980–2006	Entire Danish population. COPD identified through hospital admission codes or COPD as cause of death	*Age* <30–7% 30–59–54% 60–79 –35 % >80–3% *Sex* 55% male *COPD severity* NR *Current smokers* NR *History of CVD* NR	Discharge diagnosis of MI or cause of death from Danish Causes of Death Registry listed as MI	HR 1.26 (1.25–1.27)	Age, sex, Danish ancestry, geographical residency (rurality), and level of education
Yin *et al*^ 32^	Cohort of all residents of Sweden aged over 18, July 2005- December 2008.	51 348 COPD patients identified by diagnostic codes from patient records. 6 743 342 non-COPD patients.	**Those with no previous MI or stroke** *Age* Mean 71.1 *Sex* 44.3% male *COPD severity* NR *Current smokers* NR *History of CVD* No previous MI **Those with previous MI** *Age* Mean 69.2 *Sex* 58.4% male *COPD severity* NR *Current smokers* NR *History of CVD* All had previous MI	Diagnostic code for MI, or primary cause of death listed as MI	*No previous MI or stroke:* HR 1.47 (1.41–1.55)* Previous MI: HR 1.33 (1.23–1.43)*	Age, sex, socioeconomic status, use of cardiovascular and respiratory medicines.

*Data from personal communication (Magnus Back. Email communication. 18/08/2014).

COPD, chronic obstructive pulmonary disease; CVD, cardiovascular disease; FEV1, forced expiratory volume in 1 s; ICD, International Classification of Diseases; IRR, incidence rate ratios; MI, myocardial infarction.

**Table 2:** Characteristics of included studies—risk of MI associated with AECOPD

Study	Design and setting	Population	Characteristics	AECOPD definition	MI definition	Risk periods	Risk estimate (95% CI)
Donaldson *et al*^9^	Self-controlled case series in The Health Improvement Network, 2003–2005	426 patients with COPD and MI during study period. COPD defined using Quality and Outcomes Framework codes	*Age*: Median 74 years (IQR, 67 to 80) *Sex*: 61% male *Current smokers*: NR *COPD severity*: *Median FEV_1_% predicted*: 55.9% (IQR, 43% to 73%) *History of CVD*: NR	Three definitions used: Prescription of oral steroids Prescription of pre-specified antibiotic Prescription of pre-specified antibiotic and prescription of oral steroid	Diagnostic code for MI in primary care record	1–5 days, 6–10 days, 11–15 days, 16–49 days, and 1–49 days	Antibiotics and steroids definition: *1–5 days*: IRR 2.27 (1.10 to 4.70) *6–10 days*: IRR 1.74 (0.80 to 4.0) *11–15 days*: IRR 0.90 (0.30 to 2.90) *16–49 days*: IRR 0.83 (0.50 to 1.40) *1–49 days*: IRR 1.11(0.70 to 1.70)
Halpin *et al*^21^	Secondary analysis of patients in UPLIFT RCT	3 512 Patients with COPD who survived at least their first AECOPD. COPD defined as age ≥40 years, smoking history ≥10 pack-years, FEV_1_ ≤70% predicted, and FEV_1_/FVC ≤70%	*Age*: Mean 64 (SD, 8) *Sex*: 74% male COPD severity GOLD stage II 43% GOLD stage III 46% GOLD stage IV 9%Mean FEV_1_% predicted 38% (SD, 12) *Current smokers*: 29%*History of CVD*: NR	Increase in or new onset of more than one of: cough, sputum, sputum purulence, wheezing or dyspnoea; lasting three or more days and requiring treatment with an antibiotic or oral steroid. Data on timing of AECOPD collected at study visits	MI ascertained during RCT follow-up and recorded as a serious adverse event	30 days after AECOPD, compared to 30 days before AECOPD	IRR 13.04 (1.71 to 99.7)

AECOPD, acute exacerbation of COPD; COPD, chronic obstructive pulmonary disease; CVD, cardiovascular disease; GOLD, Global Initiative for Chronic Obstructive Lung Disease; FEV1, forced expiratory volume in 1 s; FVC, forced vital capacity; IRR, incidence rate ratios; MI, myocardial infarction; RCT, randomised control trial.

**Table 3.** Characteristics of included studies – risk of death after MI

Study	Design and setting	Population	COPD patient characteristics	Maximally adjusted estimate for mortality (95% CI)	Factors adjusted for
Andell *et al*^ 10^	Cohort study within the Swedish SWEDEHEART registry between 2005–2010.	Consecutive patients admitted to Swedish coronary care units. COPD diagnosis ascertained through linkage to the Swedish National Patient Registry.	*Age* Mean 75 years (SD, 9) *Sex* 54% male *COPD severity* NR *Current smokers* 32.9% *History of CVD* Prior MI 13.7% Prior HF 20.2%	Mortality at one year: HR 1.14 (1.07–1.21)	Age, sex, smoking, comorbidity (previous MI, previous stroke, heart failure, renal failure, hypertension, diabetes, peripheral artery disease, cancer and previous bleeding), in hospital treatment and discharge medications (heparin, fondaparinux, dalteparin, enoxaparin, glycoprotein IIb/IIa inhibitors, angioplasty, coronary stenting, β-blockers, aspirin, clopidogrel, prasugrel, calcium channel blockers, digoxin, diuretics, statins, nitrates and warfarin).
Behar *et al*^ 22^	Cohort study in Israel between 1981–1983	2276 consecutive patients surviving an MI after admission to 13 coronary care units. Patients with a history of chronic bronchitis or chronic airways obstruction and clinical and/or radiographic findings compatible with COPD during hospitalisation for MI were included.	*Age* Mean 66.8 years (SD, 9.7) *Sex* 79.3% male *COPD severity* NR *Current smokers* 43.3% *History of CVD* Prior MI—28.8% Prior angina—55.4%	*Unadjusted*:* In –hospital RR 1.39 (1.16–1.67) 1 year RR 1.34 (1.16–1.55) 5 years RR 1.28 (1.18–1.40)	
Bursi *et al*^ 23^	Cohort study of the population in the Rochester Epidemiology project involving residents in Olmsted County, Minnesota from 1979 to 2007	Local residents in Olmsted County. MI ascertained from medical records compatible with ICD criteria. Information on COPD was also obtained from ICD codes.	*Age* Mean 73 years (SD, 11) *Sex* 59% male *COPD severity* NR *Current smokers* 35% *History of CVD* Those with prior CVD excluded	HR 1.30 (1.10 to 1.54), mean follow up 4.7 years.	Age, sex, smoking, hypertension, MI type (STEMI/non-STEMI), creatine kinase level, killip class, reperfusion treatment in hospital, use of drugs on discharge (β-blockers, ACEi, diuretics)
Dziewierz *et al*^ 24^	Cohort study within Krakow Registry of ACS in February 2005-March 2005 and December 2005-January 2006	1414 patients with MI admitted to hospital in Krakow, Poland. Those with a previous history of COPD and current treatment with a steroid or bronchodilator were classified as COPD patients.	*Age* Mean 71.8 years (SD, 11) *Sex* 62% male *COPD severity* NR *Current smokers* 40.7% *History of CVD* MI 34.6% Angina 80.2% HF 30.9%	HR 2.15 (1.30–3.55)	Age, sex, BMI, diabetes, hypertension, hyperlipidaemia, prior angina, prior MI, prior heart failure, left ventricular ejection fraction, prior PCI, prior CABG, prior stroke or transient ischaemic attack, smoking status, peripheral arterial disease, chronic renal insufficiency, parameters on admission (chest pain, cardiogenic shock, heart rate, systolic blood pressure, diastolic blood pressure), time from chest pain onset to admission and type of MI (STEMI or NSTEMI)
Enriquez *et al*^ 11^	Cross sectional study of National Cardiovascular Data Registry in the USA between January 2008 and December 2010	158 890 patients admitted to one of 445 sites with an MI. COPD patients had a history of COPD or were using long term inhaled or oral β-agonists, inhaled anti-inflammatory agents, leukotriene receptor antagonists or inhaled steroids.	*Age* STEMI—median 66 years nSTEMI—median 70 years *Sex* STEMI—60.4% male nSTEMI—57.5% male *COPD severity* NR *Current smokers* STEMI—57.0% nSTEMI—41.9% *History of CVD* *STEMI* Prior MI—29.7% Prior CHF—15.3% *nSTEMI* Prior MI—39.% Prior CHF—33.3%	*In-hospital mortality* STEMI OR 1.05 (0.95–1.17) Non-STEMI OR 1.21 (1.11–1.33)	Age, serum creatinine, systolic blood pressure, troponin elevation, heart failure or cardiogenic shock at presentation, ST-segment changes, heart rate and prior peripheral arterial disease.
Hadi *et al*^ 15^	Cross sectional study of patients hospitalised with ACS in May 2006 and January 2007 to June 2007 in six Middle Eastern countries	8169 consecutive patients in the Gulf RACE registry presenting with ACS at 65 centres across six countries. COPD patients were identified from 1) medical records or 2) use of COPD medicines.	*Age* Median 64 (IQR, 56–71) *Sex* NR *COPD severity* NR *Current smokers* 38.7% *History of CVD* Prior MI—34.8% Prior angina – 54.4%	*In hospital mortality:* OR 0.40 (0.20–1.24)	Age, sex, cardiogenic shock, use of thrombolysis, use of aspirin, use of β-blocker, use of ACEi
Hawkins *et al*^ 25^	Cohort study of patients with acute MI enrolled in VALIANT trial	Patients with MI complicated by LVSD and HF. COPD was identified by a questionnaire completed by trial site investigators.	*Age* Mean 68.1 (SD, 9.9) *Sex* 71.1% male *COPD severity* NR *Current smokers* 42.0% *History of CVD* Prior MI—39.9% Prior angina—46.1% Prior HF—27.3%	HR 1.14 (1.02–1.28)	Age, heart rate, systolic and diastolic blood pressure, weight, baseline creatinine, smoking status, diabetes, dyslipidaemia, hypertension, killip classification, anterior MI, new lower bundle branch block, thrombolytic therapy, primary PCI, coronary artery bypass graft, history of heart failure, atrial fibrillation, previous MI, angina, previous stroke, peripheral arterial disease, renal insufficiency, alcohol abuse, country of enrolment, beta blocker use, randomised treatment
Kjoller *et al*^ 13^	Cohort study of consecutive patients recruited 1–6 days after an MI	Danish hospitals between May 1990 and July 1992 as part of TRACE study. COPD was identified using either 1) medical records or 2) patient report in addition to use of COPD medicines	*Age* Median 70.5 (5–95 percentiles, 50.7–83.5) *Sex* 68.2% men *COPD severity* NR *Current smokers* 60.0% *History of CVD* Previous MI—25.1% Previous angina—43.9 Previous CHF—28.2%	*Cohort entry to 30 days:* HR 0.89 (0.68–1.11) *Cohort entry to 7 years:* HR 1.15 (1.04–1.28)	Age, sex, BMI, hypertension, diabetes, smoking status, previous angina, wall motion index, angina, history of CHF, new CHF, atrial fibrillation, bundle branch block, wall motion index, use of thrombolytic therapy
Quint *et al*^ 26^ (abstract)	Cohort study of patients admitted after a first MI using data from the UK CALIBER database	8 065 patients admitted to UK hospitals with a first MI between Jan 2003-Dec 2008. COPD was identified using primary care records.	*Age* NR *Sex* NR *COPD severity* NR *Current smokers* NR *History of CVD* NR	*Mortality up to 7 years:* HR 1.37 (1.23–1.52)	Age and sex
Raposeiras *et al*^ 12^ (abstract)	Cross sectional and cohort study of patients with ACS	4 497 consecutive patients admitted to Spanish hospitals for ACS. The ascertainment method for COPD was unclear.	*Age* NR *Sex* NR *COPD severity* NR *Current smokers* NR *History of CVD* NR	*In-hospital death* OR 1.04 (1.03–1.04) *Follow up mortality* HR 1.69 (1.41–2.03), median follow up 3.1 years	GRACE score β-blocker therapy
Rha *et al*^ 33^ (abstract)	Case control study in Korea AMI registry from 2005 to 2007	AMI patients in KAMIR	*Age* Mean 71.7 (SD 10.0) *Sex* NR *COPD severity* NR *Current smokers* NR *History of CVD* NR	*Mortality at 8 months* OR 2.69, 95% CI could not be calculated from reported information.	Unadjusted
Salisbury *et al*^ 27^	19 centre prospective study of patients presenting with MI in a cohort study	MI patients in PREMIER study restricted to patients discharged alive after MI. Patients were considered to have COPD if they had a documented history of obstructive pulmonary disease (COPD or asthma) or had therapy specific for obstructive pulmonary disease.	*Age* Mean 64.5 (SD, 12.4) *Sex* 61.8% male *COPD severity* NR *Current smokers* 37.6% *History of CVD* Previous MI—29.7% Previous HF—24.3%	*Mortality up to 1 year* HR 2.00 (1.44–2.79)	Age, gender, race, avoidance of health care due to cost, smoking, diabetes, hypertension, CHF, ejection fraction, previous CVD, MI diagnosis type, new onset HF after MI, diseased vessels on angiogram, enrolling site, percentage of MI quality of care indicators of the centre, treatment type
Stefan *et al*^ 14^	Cross sectional study with follow up of patients hospitalised with AMI at greater Worcester, Massachusetts between 1997–2007	Patients hospitalised with AMI in greater Worcester, Massachusetts medical centres. COPD patients were identified by previous mention of clinical or radiographic evidence for COPD in their medical record.	*Age* Mean 74 years *Sex* 52.4% male *COPD severity* NR *Current smokers* 27.3% *History of CVD* Prior angina—22.3% Prior HF—38.6%	*In hospital*: OR 1.25 (0.97–1.34) *30 day mortality*: OR 1.31 (1.10–1.58)	Age, sex, year of hospitalisation, history of CVD, history of renal failure, type of MI (STEMI/non-STEMI), length of stay, smoking status used in secondary analysis

*Calculated from reported data.

AMI, acute myocardial infarction; BMI, body mass index; CABG, coronary artery bypass grafting; COPD, chronic obstructive pulmonary disease; CVD, cardiovascular disease; HF, heart failure; LVSD, left ventricular systolic dysfunction; MeSH, Medical Subject Headings; MI, myocardial infarction; NR, not reported; PCI, percutaneous coronary intervention; NSTEMI, non–ST-segment elevation myocardial infarction; STEMI, ST-segment elevation myocardial infarction.

All of the included studies which investigated risk of MI in people with COPD used data from either routine clinical or administrative databases. COPD was defined using diagnostic codes; these varied between COPD diagnosis in primary care, outpatient departments, hospital admission or discharge codes, and cause of death codes. Three studies also required that patients with COPD had been prescribed COPD medicines. One of the studies, Rodriguez *et al*,^19^ included only patients with a recent diagnosis of COPD and followed up to 5 years after this diagnosis to identify MI. Only one study^3^ reported a summary of COPD severity, and only two reported prevalence of current smokers. Four studies reported a cohort analysis only. Two studies^4^ ^19^ reported a cohort analysis as well as a case–control analysis. One study reported the results of a cohort analysis and an analysis of period prevalence. One study^20^ compared rates of MI in patients with COPD to standardised populations rates of MI.

Two studies^9^ ^21^ were identified which investigated the risk of MI associated with AECOPD. Both studies defined risk periods after the onset of AECOPD and used within person designs to compare the risk to a baseline period.

Nine studies reported mortality for patients with COPD after an MI compared to patient without COPD. Five studies^11^ ^12^ ^14^ ^15^ ^22^ reported a comparison of in-hospital mortality after an MI between patients with COPD and patient without COPD. Eight studies^10^ ^12^ ^13^ ^23–27^ used a time-to-event analysis to investigate death after discharge from a hospital admission for MI.

### Risk of bias assessment

**Figure 2:**
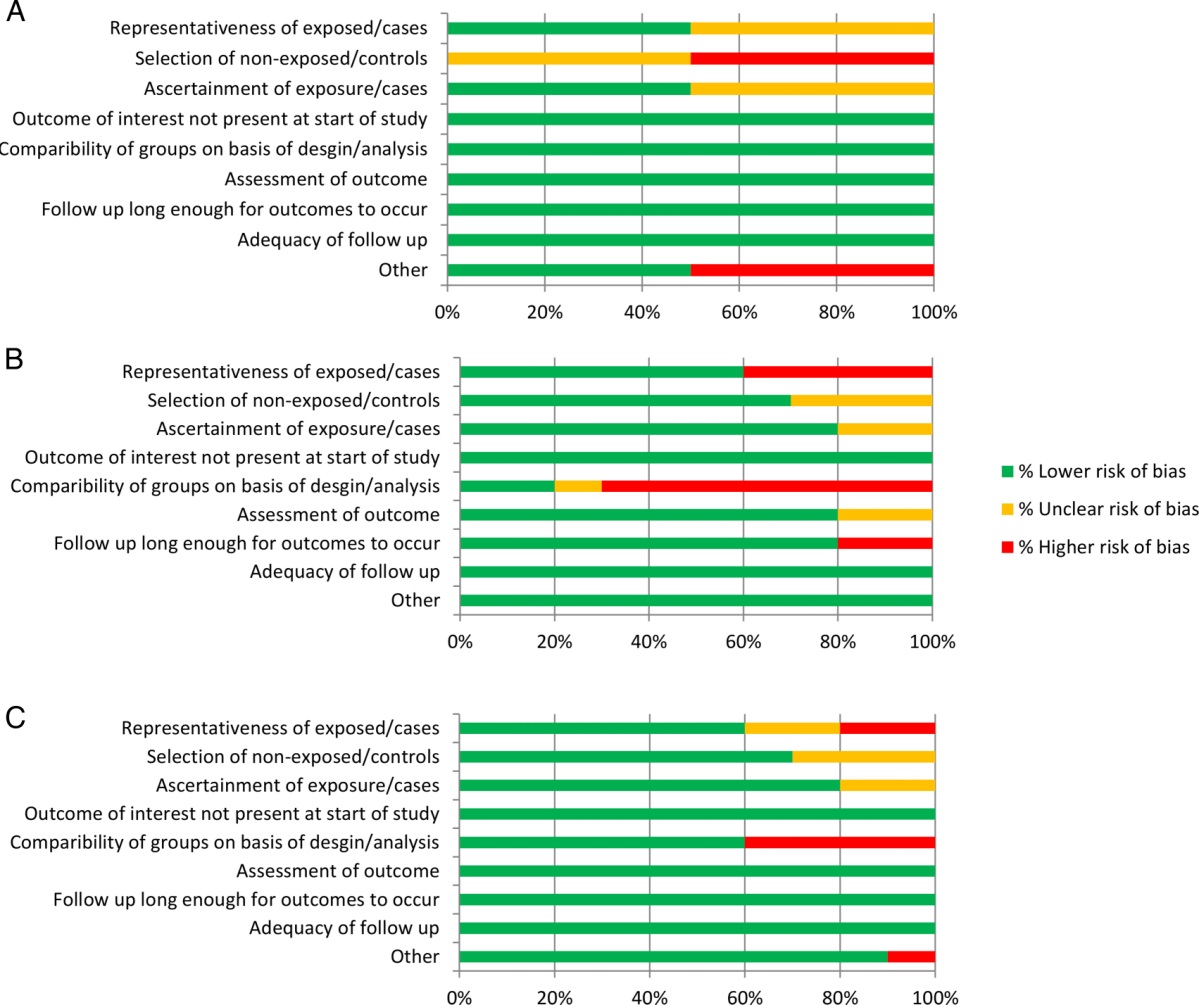
Summary of risk of bias for risk assessments for: A studies investigating risk of MI associated with COPD; B studies investigating risk of MI associated with AECOPD; and C studies investigating risk of death following MI in people with COPD. AECOPD, acute exacerbation of COPD; COPD, chronic obstructive pulmonary disease; MI, myocardial infarction.

The proportion of studies (or analyses, where appropriate) which were assessed as having either lower, unclear or higher risk of bias for each of the research questions is presented in figure 2. Detailed results from the risk of bias assessment for individual studies are presented in the appendix.

### Risk of MI in people with COPD

**Figure 3:**
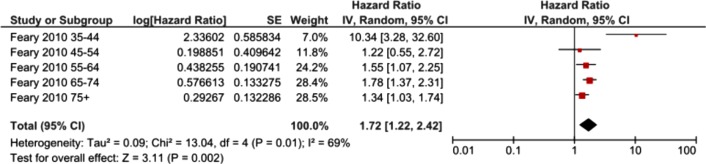
Forest plot showing risk of MI associated with COPD in cohort studies which adjusted for smoking status. CIs may vary slightly from those quoted in tables due to transformation during meta-analysis. COPD, chronic obstructive pulmonary disease; MI, myocardial infarction.

**Figure 4:**
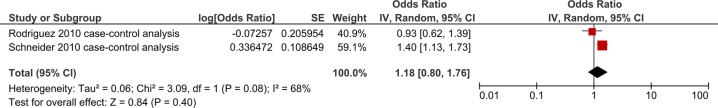
Forest plot showing risk of MI associated with COPD in case-control studies which are adjusted for smoking status. CIs may vary slightly from those quoted in tables due to transformation during meta-analysis. COPD, chronic obstructive pulmonary disease; MI, myocardial infarction.

**Figure 5:**
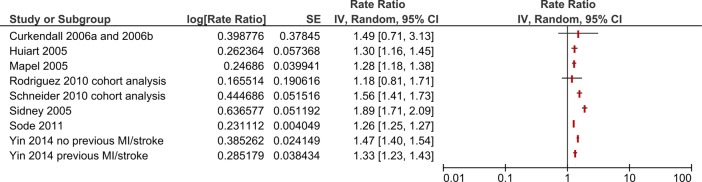
Forest plot showing risk of MI associated with COPD in cohort studies which did not adjust for smoking status. CIs may vary slightly from those quoted in tables due to transformation during meta-analysis. COPD, chronic obstructive pulmonary disease; MI, myocardial infarction.

Of nine included studies, eight found a higher risk of MI in patients with COPD compared to patient without COPD. Six studies estimated the ratio of incidence rates of MI in patients with COPD compared to patient without COPD. Five studies^4^ ^19^ ^20^ ^28^ ^29^ estimated this for all MIs, this ranged from IRR 1.18 (95% CI 0.81 to 1.71) to 5.31 (4.54 to 6.21). One study^2^ ^30^ estimated the IRR for hospitalisation due to MI (IRR 1.49, 95% CI 0.71 to 3.13) and fatal MIs (1.51, 1.14 to 2.01). Two studies^31^ ^32^ estimated the ratio of hazard of MI in patients with COPD compared to patient without COPD one study estimated this to be HR 1.26 95%, CI 1.25 to 1.27, while the other study estimated this to be HR 1.47 (1.41 to 1.55) for those with no previous MI, and HR 1.33 (1.23 to 1.43) for those with a previous MI. One study^2^ ^30^ estimated the ratio of odds of period prevalence over 5 years of acute MI in patients with COPD compared to patient without COPD (OR 1.61, 95% CI 1.43 to 1.81). Only one^3^ of the included cohort studies compared risk of MI in people with COPD and people without COPD adjusted for smoking status. This study reported results stratified by age groups. Meta-analysis of these results showed an increased risk of MI for people with COPD (HR 1.72, 95% CI 1.22 to 2.42) (figure 3). Two of the included case–control studies adjusted for smoking status. Meta-analysis of these results did not show an increased risk of MI for people with COPD (OR 1.18, 95% CI 0.80 to 1.76) (figure 4). Meta-analysis was not conducted for the studies which did not adjust for smoking as heterogeneity was too high (I^2^=93%). These results are graphically summarised in figure 5.

Some studies investigated whether the effect of COPD on the risk of MI was different in terms of age and severity of airflow obstruction. Feary *et al*^3^ found that the effect of COPD on risk of MI was higher in the 35–44 year age group (HR 10.34, 95% CI 3.28 to 32.6) compared to older age groups (45–54 years: HR 1.22 (95% CI 0.55–2.74), 55–64 years: HR 1.55 (95% CI 1.07 to 2.26), 65–74 years: HR 1.78 (95% CI 1.37 to 2.31), ≥75 years: HR 1.34 (95% CI 1.03 to 1.73)). Sidney 2005^29^ reported similar findings; the effect of COPD on risk of MI was higher in those who were aged 40–64 years (HR 2.43, 95% CI 1.98 to 2.98) compared to those who were aged over 64 years (HR 1.73, 95% CI 1.54 to 1.94). Schneider *et al*^4^ investigated the risk of MI by sub-group of COPD severity. They found that the effect of COPD on the risk of MI was greater in those with severe COPD (OR 3.00, 95% CI 1.53 to 5.86) compared to those with moderate COPD (OR 1.30, 95% CI 1.04 to 1.62) or mild COPD (OR 1.79, 95% CI 1.12 to 2.86).

### Risk of MI associated with AECOPD

**Figure 6:**
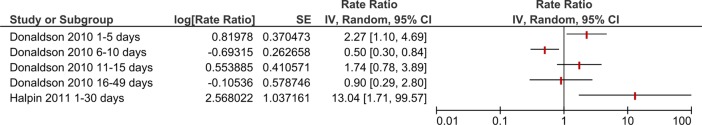
Forest plot showing risk of MI associated with acute exacerbations of COPD. CIs may vary slightly from those quoted in tables due to transformation during meta-analysis. COPD, chronic obstructive pulmonary disease; MI, myocardial infarction.

Donaldson 2010^9^ conducted a self-controlled case series using data from The Health Improvement Network (THIN). They used prescription of antibiotics and steroids in patient with COPD to identify AECOPD, and report an increased risk of MI in the 1–5 days following the onset of AECOPD (IRR 2.27, 95% CI 1.10 to 4.70). No difference in the risk of MI was found for the period 6–49 days, or at any time point when the alternative definitions of AECOPD of prescription of steroids alone or antibiotics alone were used. Halpin *et al*^21^ reported a secondary analysis of the UPLIFT trial, which was an RCT comparing inhaled tiotropium and placebo in patients with COPD with a primary outcome of reduction in FEV_1_ decline. Time to first AECOPD was a secondary outcome. AECOPD were identified using a symptom-based definition and were reported to trial staff at regular study visits. Data on MI were collected as serious adverse events. This study found that compared to the 30 days prior to AECOPD, risk of MI in the 30 days following AECOPD was increased (IRR 13.04; 95% CI 1.71 to 99.7). These results are graphically summarised in figure 6. Owing to different exposure time periods, the results for within person studies investigating the risk of MI associated with AECOPD were not pooled in meta-analysis.

### Risk of death after MI in people with COPD

**Figure 7:**
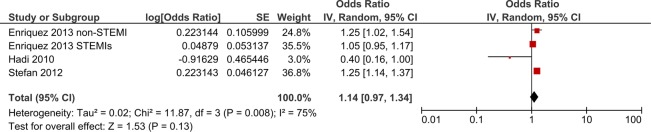
Forest plot showing risk of in-hospital death following MI for patients with COPD compared to patients without COPD. CIs may vary slightly from those quoted in tables due to transformation during meta-analysis. COPD, chronic obstructive pulmonary disease; MI, myocardial infarction.

Of the studies investigating differences in in-hospital mortality after an MI, two^12^ ^22^ found an increased risk of mortality for patients with COPD (RR 1.39, 95% CI 1.16 to 1.67 (unadjusted); and OR 1.04, 95% CI 1.03 to 1.04). Two studies^14^ ^15^ did not find evidence for increased in-hospital mortality for patients with COPD (OR 0.40, 95% CI 0.20 to 1.24; OR 1.25, 95% CI 0.97 to 1.34). One study^11^ reported results split by type of MI: it did not find an increased in-hospital mortality for patients with COPD after a STEMI (OR 1.05, 95% CI 0.95 to 1.17), but did find increased in-hospital death for patients with COPD after a non-STEMI (OR 1.21, 95% CI 1.11 to 1.33). Meta-analysis of adjusted results showed weak evidence for an increased risk of in-hospital death for patients with COPD (OR 1.13, 95% CI 0.97 to 1.31) (figure 7).

One study^14^ reported mortality at 30 days for patients with COPD compared to patient without COPD. This study found increased mortality for patients with COPD (OR 1.31, 1.10 to 1.58). Another study^33^ reported mortality at 8 months, and in an unadjusted analysis, found increased mortality for patients with COPD compared to patients without COPD (OR 2.69, 95% CI was not reported and could not be calculated). One study^22^ also found, on unadjusted analysis, that mortality was greater for patients with COPD at 1 year (RR 1.34, 95% CI 1.16 to 1.55) and 5 years (RR 1.28, 95% CI 1.18 to 1.40) after MI.

**Figure 8:**
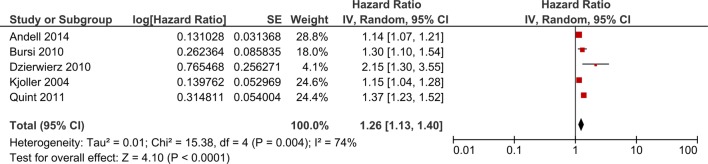
Forest plot showing risk of death after discharge following MI for patients with COPD compared to patients without COPD. Cis may vary slightly from those quoted in tables due to transformation during meta-analysis. COPD, chronic obstructive pulmonary disease; MI, myocardial infarction.

Eight studies^10^ ^12^ ^13^ ^23–27^ reported results of survival analysis of mortality during follow-up after an MI. All of the studies reported higher mortality for patients with COPD compared to patients without COPD during follow-up after discharge following an MI. HRs ranged from 1.15 (95% CI 1.04 to 1.28) to 2.15 (95% CI 1.30 to 3.55). However, one of these studies^13^ found no evidence of a difference in mortality when restricting the time period to the first 30 days following discharge (HR 0.89, 95% CI 0.68 to 1.11). Meta-analysis of studies which reported adjusted results showed an increased risk of death after discharge following MI for patients with COPD compared to patients without COPD (HR 1.26, 1.13 to 1.40) (figure 8). Four of the studies included under this question were excluded from meta-analysis for methodological^12^ ^33^ or clinical heterogeneity.^25^ ^27^

## 

## Discussion

### Main findings

Most studies which investigated the risk of MI in people with COPD found that those with COPD have higher risk of MI than people who do not have COPD; however, it is unclear how much of this increased risk is due to smoking status. The included cohort study which adjusted for smoking status showed an increased risk of MI in people with COPD, but this was not apparent in pooled analysis of the case–control studies which adjusted for smoking status. Both of the included studies which investigated the risk of MI associated with AECOPD found an increased risk of MI in the weeks following AECOPD. Most studies which investigated mortality after an MI for patients with COPD as compared to patients without COPD found that mortality after discharge was greater for those with COPD, and an increased risk of death was found on pooled analysis. However, findings on in-hospital mortality after an MI were mixed, and there was only weak evidence for increased risk of death in hospital for patients with COPD on pooled analysis.

### Limitations of included studies and future work

One common limitation among the included studies, particularly those which investigated the risk of MI associated with COPD, was missing information on smoking status. As smoking is very strongly associated with COPD and risk of MI, it is likely to be a major confounder in all studies investigating this association. All of the studies in this review which investigated this association used either clinical or administrative routine data sources. Routine data are a potentially rich source of information about huge numbers of patients. However, data on smoking are not routinely recorded in all administrative databases. Indeed, all of those studies which did not have data for smoking in this question used administrative databases. Future studies on the association between COPD and cardiovascular disease should use data sources which contain reliable information on smoking status.

Further studies should be carried out to confirm findings that AECOPD are periods of increased risk of MI for people with COPD. These studies should ensure they use validated exposure measures and are adequately powered. Possible reasons for an increased risk of MI during AECOPD include increased inflammation and the potential cardiovascular effects of the drugs used to treat AECOPD. If indeed the finding of increased risk during AECOPD is confirmed, future studies should attempt to disentangle the reasons for increased risk of MI. In addition, studies should investigate factors which might modify this relationship, such as drugs used for treatment of COPD and cardiovascular prevention. Another potential bias in studies which investigate the relationship between AECOPD and MI, which could explain some of the increased risk of MI after AECOPD, is differential misclassification of episodes of angina as AECOPD.

No studies were found which investigated the risk of MI associated with the frequent exacerbator phenotype. The frequent exacerbator phenotype may prove to be a useful characteristic for stratifying cardiovascular risk among patients with COPD. Future cohort studies of cardiovascular disease in people with COPD should, where possible, phenotype participants and investigate the relationship between exacerbator phenotype and risk of MI. Few included studies assessed the influence of severity of COPD on risk of MI; further research should investigate this relationship as well as the influence of severity of COPD on risk of death following MI.

A further limitation of several of the included studies on death following MI was availability of information on cause of death. Collection of information on cause of death in future studies would allow investigators to draw more confident conclusions about the reasons for increased risk of death following MI for people with COPD.

### Strengths and limitations of this review

This review benefitted from using a comprehensive search strategy which covered several bibliographic databases. As the relationship between AECOPD and MI has not been extensively studied, the inclusion criteria for this research question were kept purposively broad. This allowed all information pertaining to this relationship to be included in the evidence synthesis. One potential limitation of systematic reviews is publication bias. The potential for publication bias was highest for the review of outcomes after MI. In order to reduce the risk of this bias, we only included studies which specifically investigated the risk of COPD on MI rather than several different potential prognostic factors, as studies which investigated several factors which did not find an association between COPD and MI may not have reported this in the abstract or even in the text. Owing to clinical and statistical heterogeneity, meta-analysis could only be conducted for some of the research questions. Where meta-analysis was conducted, statistical heterogeneity was generally high, and this may limit the generalisability of pooled estimates.

## Conclusions

There is good evidence of an increased risk of MI in people with COPD; however, it is unclear to what extent this association is due to smoking status.

There is some evidence that among people with COPD, AECOPD represent periods of increased risk of MI. However, further larger studies using validated exposure methods are needed to support this finding.

There is weak evidence that in-hospital mortality is higher for people with COPD after an MI. There is good evidence that postdischarge mortality after an MI is higher for people with COPD.
